# Tyrosine Kinases in *Helicobacter pylori* Infections and Gastric Cancer

**DOI:** 10.3390/toxins11100591

**Published:** 2019-10-11

**Authors:** Bianca E. Chichirau, Sebastian Diechler, Gernot Posselt, Silja Wessler

**Affiliations:** 1 Department of Biosciences, Paris-Lodron University of Salzburg, 5020 Salzburg, Austria; bianca.chichirau@sbg.ac.at (B.E.C.); sebastian.diechler@sbg.ac.at (S.D.); gernot.posselt@sbg.ac.at (G.P.); 2 Cancer Cluster Salzburg, Department of Biosciences, Paris-Lodron University of Salzburg, 5020 Salzburg, Austria

**Keywords:** *Helicobacter pylori*, tyrosine kinases, c-Abl, CagA, Src family kinases, EGFR, c-Met

## Abstract

*Helicobacter pylori* (*H. pylori*) has been identified as a leading cause of gastric cancer, which is one of the most frequent and malignant types of tumor. It is characterized by its rapid progression, distant metastases, and resistance to conventional chemotherapy. A number of receptor tyrosine kinases and non-receptor tyrosine kinases have been implicated in *H. pylori*-mediated pathogenesis and tumorigenesis. In this review, recent findings of deregulated EGFR, c-Met, JAK, FAK, Src, and c-Abl and their functions in *H. pylori* pathogenesis are summarized.

## 1. Introduction: Tyrosine Kinases in Health and Disease

The human ‘kinome’ consists of 535 protein kinases and represents the largest protein family in humans [1]. Approximately 90 of these protein kinases belong to the family of tyrosine kinases, which can be sub-classified into receptor tyrosine kinases (RTK) and non-receptor tyrosine kinases (nRTK). In normal tissues, tyrosine kinases control a wide range of cellular functions including differentiation, proliferation, survival, apoptosis, motility, and cell adhesion. However, deregulated tyrosine kinase activities in response to epigenetic modifications, gene amplification or mutations have been implicated in the development of a number of human diseases, in particular inflammation, diabetes, and cancer [1,2].

Although the incidence in Europe has declined in the last 30 years, gastric cancer is still a leading cause of cancer-related deaths [3]. Endoscopic resection represents a curative strategy for early gastric neoplastic lesions, but is associated with unfavorable prognosis at advanced stages of gastric cancer [4]. The upregulation of several RTKs and nRTKs in gastric cancer has been reported, suggesting that pharmacological targeting of tyrosine kinases might be beneficial and might increase the survival time of patients [5]. High-throughput analyses have revealed that epidermal growth factor receptor (EGFR), fibroblast growth factor receptor (FGFR), platelet-derived growth factor receptor (PDGFR), the hepatocyte growth factor (HGF) receptor c-Met, and vascular endothelial growth factor receptor (VEGFR) are expressed at high levels in gastric cancer compared to normal tissues [6], suggesting that RTKs play a primary role in carcinogenesis. Additionally, epigenetic changes in the *egfr* locus have been observed in gastric cancer and it has been suggested that *egfr* promoter hypermethylation could serve as a potential biomarker for gastric cancer status and progression [7]. Similar observations have been made for a number of nRTKs. Elevated mRNA and protein expression levels of Src and Lyn have been detected in gastric tumors that may have a role in invasiveness and metastasis [8]. Accordingly, targeted therapy against deregulated tyrosine kinases in gastric cancer is being intensively discussed.

The paradigm of targeted therapy directed against kinases in cancer is the tyrosine kinase inhibitor (TKI) Gleevec (STI-571, Imatinib, Novartis). Gleevec mainly blocks kinases of the Abl family, but also PDGFR and c-Kit, all of which are implicated in chronic myelogenous leukemia (CML), acute lymphocytic leukemia (ALL), and gastrointestinal stromal tumors (GIST) [9]. The effectiveness of Gleevec in the treatment of CML is of particular importance, since CML is characterized by a gene fusion of breakpoint cluster region and c-Abl (BCR-Abl) that results in a constitutively active kinase and uncontrolled proliferation of hematopoietic stem cells. The fusion protein BCR-Abl is produced by the genomic translocation [t(9;22)(q34;q11)], known as the Philadelphia chromosome. Gleevec binds to BCR-Abl, blocks its kinase activity, and thus halts proliferation and induces apoptosis in CML cells [9]. Motivated by this breakthrough, a number of TKIs have been developed, e.g., gefitinib and erlotinib (both block EGFR) for the treatment of lung cancer. However, subsequent TKIs have been unable to replicate the great success of Gleevec, although a significant increase in the survival time of patients has been observed in tests of small cohorts of patients [10,11,12].

Gastric cancer is strongly associated with the human pathogen *Helicobacter pylori* (*H. pylori*) which triggers a cascade of inflammation-driven carcinogenesis. Chronic infection with *H. pylori* provokes conditions such as acute and chronic gastritis, ulcer diseases, mucosa-associated lymphoid tissue (MALT) lymphoma, and gastric cancer. The bacterium has therefore been classified as a group-I carcinogen by the WHO [13]. The current model of *H. pylori*-dependent gastric cancer development follows a sequence of chronic gastritis leading to atrophy and intestinal metaplasia and finally dysplastic changes and gastric cancer [14].

RTK expression has been found to be highly deregulated in transformed gastric cancer tissues. However, *H. pylori* directly affects RTK gene expression to a limited extent. Expression analyses of *egfr* in *H. pylori*-infected patients have not revealed any clear pattern and show only minor alterations. In the literature, EGFR levels are reported to be regulated in both directions or remain unchanged in biopsies obtained from *H. pylori*-positive individuals [15,16,17,18], whereas cell culture models suggest an upregulation of EGFR [19,20,21,22]. c-Met expression has also been shown to increase in gastric epithelial cell lines after *H. pylori* exposure, but no significant difference was found in *c-met* expression in tumor tissues from *H. pylori*-positive and -negative patients [23]. Expression of VEGFR decreased slightly in human umbilical vein endothelial cells (HUVECs) upon infection with *H. pylori* [24], whereas in another study, *H. pylori* urease stimulated VEGFR expression in human microvascular endothelial cells (HMEC-1) [25]. In summary, these data suggest that overexpression of RTKs in gastric cancer occurs late in the transformation process and is largely independent of *H. pylori.* Nevertheless, neoplastic cancer cells display deregulated expression and accumulation of mutations in RTKs. According to the Cancer Genome Atlas (TCGA) project, tumor protein p53 (*TP53*) and myeloid/lymphoid or mixed-lineage leukemia 4 (*MLL4*) mutations occur with the highest frequencies in gastric cancer, but gene alterations of members of the human epidermal growth factor receptor family (*HER3*, *ERBB4* and *EGFR*) are also prevalent in gastric cancer and are considered to be important driver mutations [26].

### 1.1. H. pylori Utilizes Specific Virulence Factors to Control a Complex Network of Tyrosine Kinases

**Figure 1:**
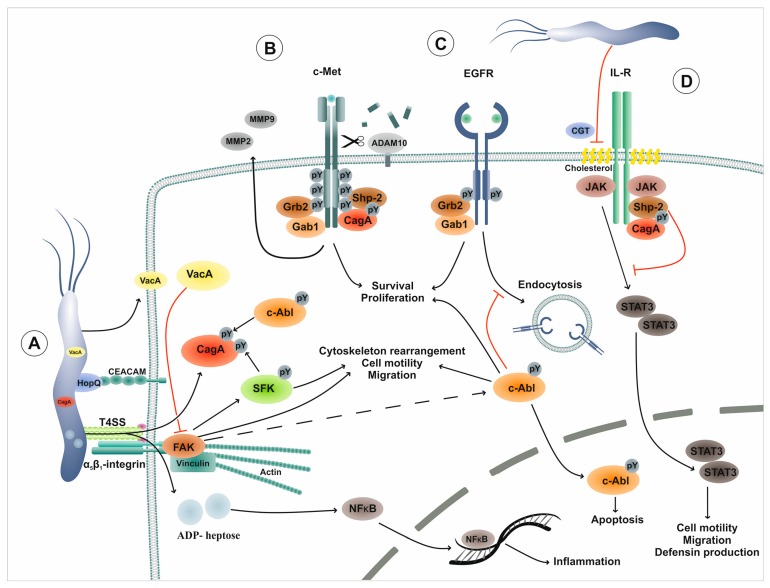
RTK and nRTK networks in *H. pylori* pathogenesis. (**A**) *H. pylori* expresses adhesins (e.g., HopQ) and secretes virulence factors (e.g., VacA) and T4SS-delivered effectors such as CagA or ADP-heptose for persistent colonization of the human gastric epithelium. Integrin stimulation leads to the activation of FAK, SFK, and c-Abl, which stimulate CagA phosphorylation, cytoskeleton rearrangements, and cell motility/migration. Injection of ADP-heptose leads to a strong NF-κB-driven inflammation. (**B**) c-Met receptors are targeted via different mechanisms: CagA can substitute Gab1 adapter proteins and stimulate proliferation and pro-survival signals. Furthermore, MMP-2 and MMP-9 activities, which aid the motogenic response, are induced via c-Met-dependent signaling. Moreover, the sheddase ADAM10 is activated and targets the extracellular domain of c-Met. (**C**) *H. pylori* mediates EGFR activation and stabilizes its surface expression by the inhibition of its endocytosis and proteasomal degradation. (**D**) *H. pylori* induces JAK/STAT3 signaling during early infections, whereas the pathway is shut down during persistent colonization. CagA mediates the recruitment of the SHP2 phosphatase and prevents STAT binding via IL-R dephosphorylation. Additionally, *H. pylori* CGT facilitates cholesterol extraction from the outer cell membrane and, thus, disrupts lipid rafts which provide membranous signaling platforms for the JAK/STAT pathway. Abbr.: RTK, receptor tyrosine kinase; nRTK, non-receptor tyrosine kinase, HopQ, *H. pylori* outer membrane protein Q; VacA, vacuolating cytotoxin A; T4SS, Type IV secretion system A; CagA, cytotoxin-associated gene A; FAK, focal adhesion kinase; SFK, Src family kinase; NF-kB, nuclear factor kappa B; MMP, matrix metalloprotease; ADAM10, A Disintegrin and metalloproteinase domain-containing protein 10; EGFR, epidermal growth factor receptor; JAK/STAT3, Janus kinase/Signal Transducers and Activators of Transcription 3; SHP2, Src homology region 2 domain-containing phosphatase-2, IL-R, interleukin-receptor; CGT, cholesterol-glucosyltransferase.

Even though the expression of RTKs and nRTKs is only marginally regulated by *H. pylori*, drastic effects on kinase activities can be seen. A number of *H. pylori* virulence factors highjack signal transduction pathways and thus interfere with host cell functions. Many significantly correlate with the risk of developing *H. pylori*-dependent gastric cancer. A central disease-promoting factor of *H. pylori* is a specific type-IV secretion system (T4SS) which is encoded by the cytotoxin-associated gene (*cag*) pathogenicity island (*cag*PAI). The T4SS critically interacts with surface receptors on host cells and also delivers several bacterial products directly into the cytosol of infected cells (Figure 1A). The T4SS forms a pilus with the pilus-associated factors CagL, CagI, and CagY, which bind to integrin-β1 and integrin-β6 and facilitate the injection of bacterial factors into gastric epithelial cells [27,28,29]. These factors include the oncoprotein cytotoxin-associated gene A (CagA) [30,31], peptidoglycan [32], chromosomal DNA, and ADP-glycero-β-D-manno-heptose (ADP-heptose) [33,34,35]. ADP-heptose is a metabolite derived from lipopolysaccharide (LPS)-biosynthesis and has only recently been described as a novel T4SS effector. Importantly, ADP-heptose mediates NF-κB activation via the alpha-kinase 1 (ALPK1)-TRAF-interacting protein with FHA domain (TIFA) axis and this is a central step in the regulation of innate immune pathways [34,35]. The T4SS-mediated translocation of CagA has been well investigated and plays a major role in pathogenesis. Injection of CagA causes intense cytoskeletal rearrangements, leading to cell elongation and increased cell migration [36,37]. Recently, CagA translocation has also been found to be associated with carcinoembryonic antigen-related cell adhesion molecule (CEACAM) surface molecules, which serve as receptors for the *H. pylori* adhesin HopQ [38,39]. However, the interplay between integrins and CEACAMs is not well understood [38,39]. Integrin receptors have been suggested to induce *H. pylori*-induced kinase signaling as *H. pylori* binding triggers the activation of β1-integrin and, subsequently, of focal adhesion kinase (FAK), Src, EGFR, and HER3 (heregulin receptor 3)/ErbB3 [28] (Table 1). The nRTK FAK is an important regulator of cell adhesion, spreading, motility, differentiation, and death [40]. *H. pylori*-mediated FAK activation is characterized by phosphorylation on Y397, Y407, Y576, Y577, Y861 and Y925. The drastic morphological changes seen in *H. pylori*-infected gastric epithelial cells have been attributed to β1-integrin-mediated FAK activation [28,41,42]. Apparently, the actin cytoskeleton regulator cortactin is also involved in integrin-induced FAK activity. Cortactin tyrosine phosphorylation is lost early in infection (cf. 1.2 Src family kinases (SFK) signaling), but serine 405 and serine 418 are phosphorylated at later time points, which leads to an interaction with and activation of FAK [42]. On the other hand, phosphorylated CagA diminishes FAK phosphorylation via recruitment of the Src homology region 2 domain-containing phosphatase-2 (SHP2) [43], suggesting that CagA counterbalances β1-integrin-FAK signaling. A similar effect has been described for the *H. pylori*-secreted virulence factor vacuolating cytotoxin A (VacA), which also reduces FAK activity [44]. The RTK c-Met also takes center stage in signaling events hijacked in *H. pylori* infections. HGF is the only known ligand for c-Met and activates signaling pathways involved in proliferation, motility, migration, and invasion [45]. The HGF-c-Met interaction induces autophosphorylation of the intracellular domain, which forms a binding platform for signaling molecules, such as growth factor receptor-bound protein 2 (Grb2) and Grb2-associated-binding protein 1 (Gab1) [46] (Figure 1B). CagA has been shown to interact with the intracellular domain of c-Met, leading to the activation of ligand-independent signaling. It has been suggested that CagA acts as an adaptor protein mimicking Gab1 and recruits additional signaling proteins [47,48], such as E-cadherin, p120 catenin, or zonula occludens-1 (ZO-1) [49,50]. The resulting signaling triggers cell migration [51], CagA-mediated autophagy [52], and proliferation of primary organoid-derived cells [53,54] (Table 1). Importantly, *H. pylori* activates the matrix metalloproteases MMP-2 and MMP-9 in a c-Met-dependent fashion [55]. Both MMPs are considered important factors in the motogenic response to *H. pylori*, as well as in metastasis of gastric cancer and tumor-associated angiogenesis [56]. Additionally, c-Met ectodomain shedding has been observed as a consequence of *H. pylori*-induced activation of A Disintegrin and metalloproteinase domain-containing protein 10 (ADAM10) [57]. This might explain why soluble c-Met can be considered an important biomarker for gastric cancer [58].

### 1.2. The nRTKs c-Abl and Src Phosphorylate CagA

CagA delivery into the host cell cytoplasm is a key process in *H. pylori* pathogenesis and development of gastric cancer [70,71]. After translocation, CagA rapidly becomes tyrosine-phosphorylated, which is a prerequisite for the strongly elongated cell morphology [72,73]. The C-terminal part of CagA contains a variable number of EPIYA motifs (Glu-Pro-Ile-Tyr-Ala), which have been identified via mass spectrometry and site-directed mutagenesis as unique CagA phosphorylation sites [72,73,74]. Four distinct EPIYA motifs have been described based on their surrounding sequence, namely EPIYA -A, -B, -C and -D, and these can be assigned to the geographic regions from where the bacterial strains were isolated [75]. EPIYA-A and -B motifs are present in all strains, whereas EPIYA-C is mainly conserved in Western isolates and replaced with EPIYA-D in strains from East Asian countries [75,76]. The number of EPIYA motifs varies from one to seven, with an average of three EPIYA segments present in the majority of strains [77].

Upon infection of gastric epithelial cells with *H. pylori*, CagA is phosphorylated by two families of nRTKs, SFKs and c-Abl, in a highly time-coordinated manner (Table 1). Both SFKs and c-Abl are implicated in processes ranging from cytoskeleton rearrangements, cell motility, and proliferation to DNA damage response and apoptotic pathways [78,79]. During earlier stages of infection (0.5–2 h), the SFK members c-Src and Lyn are rapidly activated by an unknown mechanism and directly phosphorylate CagA. Specific c-Src kinase inhibitors cause a decrease in CagA phosphorylation levels and efficiently prevent the elongation phenotype induced by *H. pylori*, whereas overexpression of c-Src in infected cells leads to an increase in CagA phosphorylation [73,80]. Interestingly, during prolonged infection (2–8 h), c-Src activity is downregulated by a negative feedback-loop involving phosphorylated CagA and its interaction with the C-terminal Src kinase (Csk), which inactivates c-Src [81]. Discontinued c-Src activity leads to the dephosphorylation of the actin-binding proteins vinculin, cortactin, and ezrin [82,83,84]; however, CagA tyrosine phosphorylation is maintained by activated c-Abl [60,61,62]. At this stage, a complex composed of phosphorylated CagA, activated c-Abl, and its substrates, Crk adaptor proteins, is formed, which is essential for the *H. pylori*-induced elongation phenotype [60,61,85]. The model of CagA phosphorylation includes a hierarchically ordered action of SFKs and c-Abl. Early c-Src activity exclusively favors the phosphorylation of EPIYA-C or -D motifs, whereas c-Abl phosphorylates EPIYA-C or -D motifs followed by EPIYA-A and -B motifs. Two-dimensional electrophoresis has revealed that only one or two motifs are phosphorylated at the same time and mutational tyrosine residue replacement in the EPIYA motifs has shown that none of the phosphorylated EPIYA motifs alone are able to initiate the characteristic cell elongation phenotype. Moreover, phosphorylation of EPIYA-A and EPIYA-C induces a similar phenotype to that induced by the presence of three intact EPIYA motifs [62]. Depending on its phosphorylation status, CagA binds different sets of signaling molecules. Tyrosine-phosphorylated CagA interacts with Crk proteins, Csk, or SHP2. Binding partners for non-phosphorylated CagA include E-cadherin, Grb2, c-Met, and ZO-1 [50], underlining the importance of the CagA phosphorylation status for the control of cellular responses to *H. pylori*. The influence of *H. pylori* on kinase networks also extends to the Janus kinase (JAK)/ Signal Transducers and Activators of Transcription (STAT) signaling axis (Figure 1 D). CagA tyrosine phosphorylation also functions as a switch between the SHP2 and JAK/STAT pathways mediated by glycoprotein gp130 receptor chains. In the presence of phosphorylated CagA, SHP2 is recruited to gp130, whereas non-phosphorylated CagA preferentially causes JAK2-mediated STAT3 activation, which leads to *c-myc* transactivation and enhanced cell migration [67]. JAK2 phosphorylation has also been observed in response to *H. pylori* lipopolysaccharide (LPS) stimulation [86,87]. However, another study has indicated that *H. pylori* activates EGFR→mitogen-activated protein (MAP) kinase→JAK/STAT signaling, leading to the synthesis of the antimicrobial human beta-defensin 3 (hBD3) [59]. Furthermore, JAK/STAT signaling has been associated with NF-κB-mediated interleukin-8 (IL-8) expression in gastric epithelial cells [69] (Table 1). *H. pylori* has been shown to interfere with JAK/STAT signaling by disrupting lipid rafts via cholesterol depletion in the outer membrane using a cholesterol-α-glucosyltransferase. This results in abrogated hBD3 production and reduced expression of interferon response genes even in the presence of high interferon gamma (IFN-γ) levels, thereby creating a protective niche for *H. pylori* [68].

**Figure 2:**
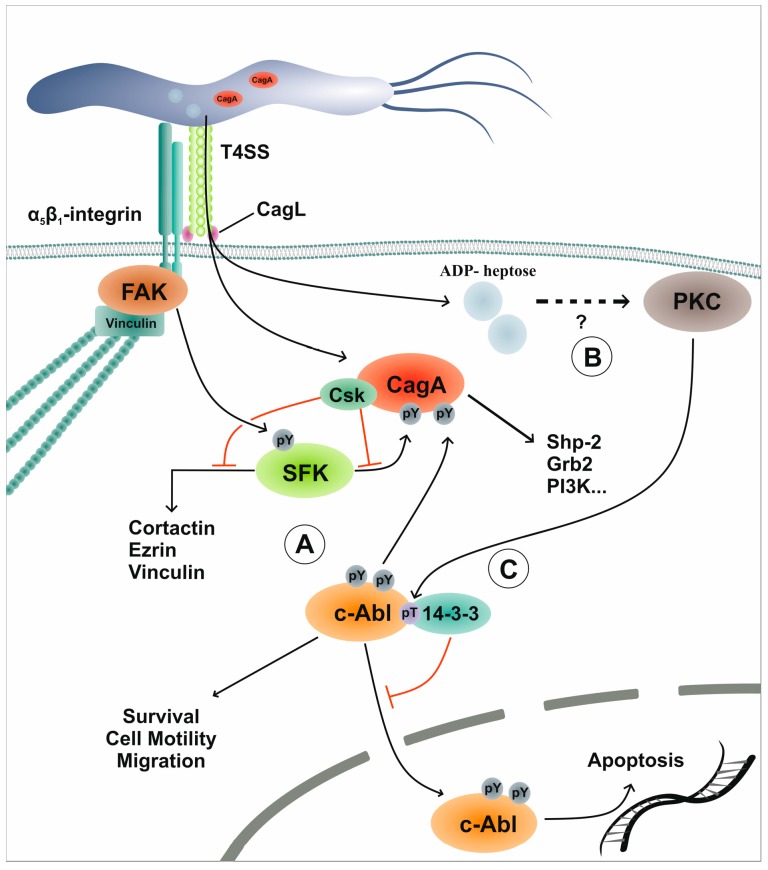
*H. pylori-*induced CagA phosphorylation by Src and Abl family kinases. (**A**) *H. pylori* injects CagA via its T4SS into human gastric epithelial cells and sequentially activates SFK and c-Abl to induce CagA phosphorylation at the EPIYA motifs. Early CagA phosphorylation triggers recruitment and activation of Csk, which feeds into a negative feedback loop, inactivates SFK, and thus causes the dephosphorylation of Src targets cortactin, ezrin, and vinculin. At this point, c-Abl maintains CagA phosphorylation. (**B**) The *H. pylori* metabolite ADP-heptose is transferred into host cells and activates PKC through a yet undescribed mechanism. PKC then phosphorylates threonine 735 of c-Abl. (**C**) Phosphorylated c-Abl^T735^ serves as a docking site for the interaction with proteins of the 14-3-3 family, which prevent nuclear shuttling of c-Abl. Cytoplasmic tethering of c-Abl forces CagA phosphorylation and increases cell survival, cell motility, and proliferation, whereas c-Abl activities in the nucleus are attenuated. Abbr.: PKC, protein kinase C; Csk, C-terminal Src kinase.

Taking into consideration that chronic infection with *H. pylori* initiates recruitment of neutrophils and lymphocytes into the gastric mucosa, a direct interaction between infiltrating cells and the bacteria is highly plausible [14,88]. Therefore, different immune cell lines (MEC-1, THP-1, U937, J774A.1) have been employed to demonstrate the successful translocation and phosphorylation of CagA in these cells upon infection [82,88]. However, the model of successive phosphorylation of CagA by SFK and Abl family kinases derived from the gastric epithelial cells does not apply to immune cells [63]. Using the MEC-1 cell line (chronic B lymphocyte leukemia cells) as an infection model, it has been shown that expression and activity of c-Src is up-regulated two hours post-infection and remains stable, whereas c-Abl activity is rapidly increased. However, the impact on the phosphorylation pattern of EPIYA motifs by the simultaneous activation of both tyrosine kinases in immune cells is not yet known. Individual inhibition of c-Src and Abl kinases moderately decreases CagA phosphorylation levels, while simultaneous inhibition of both kinases causes a complete loss of CagA phosphorylation. Moreover, cell viability assays have shown a significant reduction in *H. pylori*-mediated cell death in the presence of c-Src and Abl kinase inhibitors [63]. Therefore, SFKs and Abl family kinases contribute not only to CagA phosphorylation but also to the regulation of associated downstream signaling pathways (Figure 2).

### 1.3. c-Abl Is more than a CagA Kinase: Control of Cell Migration and Apoptosis

The regulation of c-Abl in *H. pylori*-infected cells has attracted much attention, because the c-Abl proto-oncoprotein has been strongly implicated in tumorigenesis and cancer [89]. Abl proteins share structural similarities with SFKs and are characterized by an N-terminal Src homology 2 (SH2) domain binding to phosphorylated tyrosine residues and a Src homology 3 (SH3) domain which preferentially binds to proline-rich protein sequences. The C-terminal tail of c-Abl consists of a DNA-binding domain, F- and G-actin-binding domains, nuclear localization signals (NLS), and nuclear export signals (NES), and is of high importance for the regulation of kinase activity and localization [90]. Abl kinases are activated by various factors, such as PDGFR and EGFR, substrate binding, oxidative stress, or bacterial pathogens [89,90,91]. *H. pylori* is included in the list since many studies have provided strong evidence showing that it utilizes c-Abl-CagA signaling for efficient colonization and pathogenesis [92,93]. The presence of *H. pylori* leads to a continuous activation of c-Abl and phosphorylation of the kinase on tyrosine residues 245 and 412 [60,61,64]. Moreover, kinase inhibition of c-Abl, silencing by RNA interference, or expression of a kinase-dead version cause a significant reduction in cell elongation and scattering and a decrease in late CagA phosphorylation upon *H. pylori* infection [60,61]. Furthermore, cross-talk between c-Abl and EGFR signaling has been observed. c-Abl-mediated phosphorylation of EGFR on tyrosine residue 1173 inhibits receptor endocytosis leading to an increase in EGFR surface expression in *H. pylori* infections [65,66]. This process has been shown to be dependent on CagA, but independent of CagA phosphorylation [65]. Additionally, EGFR protein turnover is slowed down by activated c-Abl via suppression of the ubiquitin ligase casitas B-lineage lymphoma (Cbl), which is involved in EGFR degradation [66] (Figure 1 C).

In *H. pylori*-infected gastric epithelial cells, c-Abl kinase activation is triggered in a CagL-dependent fashion; however, the pathways upstream of c-Abl activation are not fully understood [60,61,64]. Activation results in major conformational changes and is accompanied by phosphorylation at tyrosine 245 and tyrosine 412 [94,95]. Abl kinases have functions in manifold cellular responses, like cell migration, survival, proliferation, and cell death. These opposing effects are mainly regulated via the subcellular localization of the kinase. Depending on the cellular context and environmental signals, NLS and NES sequences regulate shuttling of c-Abl between the cytoplasm and the nucleus. In the cytoplasm, c-Abl is involved in the regulation of actin dynamics and proliferation. Accordingly, many of the identified kinase substrates (Crk proteins, cortactin, Wave, etc.) are closely associated with cell morphology, migration, and proliferation [90,96,97] (Figure 2A). In contrast, nuclear c-Abl contributes to the DNA damage response [78] and apoptosis [98,99]. The importance of the subcellular localization of c-Abl is accentuated by the action of the BCR-Abl fusion protein. In untreated cells, BCR-Abl is constitutively active solely in the cytoplasm, which leads to uncontrolled proliferation, whereas treatment with kinase inhibitors also allows shuttling of c-Abl to the nuclear compartment and cell death [100]. Therefore, a balanced nucleo-cytoplasmic transport of c-Abl is a tightly regulated process in normal cells.

The pivotal role of the subcellular localization of c-Abl in cell fate decisions implies the existence of several levels of regulation. Shuttling mediated via NLS and NES motifs has been associated with the interaction of members of the 14-3-3 protein family. The 14-3-3 proteins have been shown to interact preferentially with phosphorylated threonine 735 in the c-Abl protein (pAbl^T735^) and thereby mask the NLS motifs [101,102] (Figure 2 C). The phosphorylation of pAbl^T735^ is mediated via the dual specific kinase monopolar spindle 1 (Mps1, TTK) [102] and protein kinase C (PKC) kinases [64]. Under genotoxic or oxidative stress, c-Abl is released from 14-3-3 interaction and preferentially shuttles to the nucleus. This release is mediated via JNK-dependent 14-3-3 phosphorylation on serine 84 (as shown for 14-3-3ζ), which abrogates 14-3-3 interaction with c-Abl irrespective of Abl^T735^ phosphorylation status. Nuclear c-Abl is central to the DNA damage response and contributes to cell fate decisions between cell cycle arrest and DNA repair on the one hand, and cell death and apoptosis on the other. Nuclear c-Abl is activated via ataxia-telangiectasia mutated (ATM) and DNA-dependent protein kinase (DNA-PK) and has been shown to directly phosphorylate p73 [79]. Interaction with p53 is thought to trigger cell cycle arrest, whereas p73 and MAP-kinase signaling feed into an apoptotic signaling cascade. Interestingly, apoptotic caspases have been shown to cleave c-Abl, which results in a loss of the N-terminal NES signals and thus amplifies nuclear c-Abl accumulation and apoptosis in a positive feedback loop [103]. Further, nuclear c-Abl leads to elevated JNK activity levels in a c-Jun-dependent signaling circuit [104]. Again, JNK-dependent 14-3-3 phosphorylation might lead to a release of c-Abl from cytoplasmic tethering, resulting in nuclear accumulation. In combination, all these mechanisms reinforce nuclear c-Abl localization and activity, which sequentially could reach a point of no return and thus inevitably induce cell death.

The cytoplasmic functions of c-Abl in the course of *H. pylori* infections are well established and its roles in CagA phosphorylation and CagA-independent mechanisms have been well described [105]. However, the signaling events upstream of c-Abl activation are still unknown. Recently, we found that *H. pylori* additionally triggers the phosphorylation of threonine 735 and binding to 14-3-3, leading to cytoplasmic retention of pAbl^T735^ [64]. The phosphorylation at the critical tyrosine residues 245 and 412 during c-Abl activation is independent of threonine phosphorylation and is elicited via a distinct mechanism. Both threonine as well as tyrosine phosphorylation strictly depend on the presence of an intact T4SS. We could show that while c-Abl activation requires the T4SS structural component CagL, the threonine phosphorylation is enabled by the recently described *H. pylori* metabolite ADP-heptose, which has been shown to be injected into host cells [34,35]. In uninfected gastric epithelial cells, c-Abl activation levels are low and the subcellular distribution between cytoplasm and nucleus is in equilibrium. This picture drastically changes in response to *H. pylori* infection, when phosphorylation of c-Abl threonine 735 forces 14-3-3 binding and hence cytoplasmic localization [64]. This activation and localization pattern favors the cytoplasmic actions of c-Abl and contributes to CagA phosphorylation and cytoskeleton rearrangements leading to cell elongation and enhanced cell motility. Concomitantly, nuclear exclusion prevents apoptotic signaling and thus limits infection-induced cell death (Table 1). In contrast to the situation under conditions of genotoxic or oxidative stress, in which TTK has been identified as the kinase which phosphorylates Abl^T735^ [102], we identified PKC kinases upstream of pAbl^T735^ (Figure 2B). Syk kinase activation has been suggested to contribute to PKC activity in *H. pylori* infections in an LPS/TLR-4-mediated manner [106]. Mutational disruption of the c-Abl/14-3-3 interaction renders the anti-apoptotic action of this signaling cascade ineffective and, consequently, leads to a strong increase in apoptosis after *H. pylori* infection. In vivo, we found constant phosphorylation of the 14-3-3 interaction site Abl^T735^ in patients suffering from chronic *H. pylori* gastritis, whereas pAbl^T735^ levels were low in chemically induced gastritis and healthy individuals. In a mouse model of chronic *H. pylori* infection, the cytoplasmic c-Abl activity contributes to the aggravation of disease progression, as animals that received the c-Abl inhibitor Gleevec showed an overall milder gastric phenotype despite equal colonization levels [64].

## 2. Concluding Remarks

Tyrosine kinase inhibition has proved to be a promising strategy to support cancer therapy against non-small cell lung carcinoma (EGFR inhibitors) and leukemic malignancies (CML: ABL inhibitors; AML: FLT3 inhibitors; ALL: ABL inhibitors). TKIs of the latest generation have high target specificity and cause fewer side effects than conventional chemotherapy. Unfortunately, many TKI targets are subject to mutation and can acquire resistance to TKI inhibition. Therefore, many TKIs are used in combinatory regimens to circumvent drug resistance. In gastric cancer, changed expression levels and mutation of RTKs are implicated in tumor progression and metastasis [103]. This highlights the importance of a personalized precision medicine to specifically target deregulated signaling pathways when using TKIs. Furthermore, TKIs are not only used to directly target driver kinases: promising results have also been reported for anti-angiogenic drugs targeting VEGF signaling to prevent tumor vascularization and metastasis [5,107]. The c-Abl inhibitor Gleevec is also used in the treatment of the rare GIST cancer and some studies have suggested a role for Gleevec in combination therapy regimens targeting gastric cancer. Although kinase inhibition might be inappropriate for the treatment of non-malignant *H. pylori-*associated disease, it would be highly interesting to study disease progression in a cohort of *H. pylori*-positive CML patients being treated with TKIs. This would give important insights into the in vivo relevance of *H. pylori*-regulated kinase networks.

## Figures and Tables

**Table 1:** The role of receptor tyrosine kinases and non-receptor tyrosine kinases in *H. pylori* pathogenesis.

**RTKs**	**Function**	**Role in *Hp* Pathogenesis ^1^**	**References**
EGFR	Differentiation, growth, migration, proliferation, matrix adhesion	◾ Activated by *Hp*-integrin-β1 interaction	[28]
		◾ Activates MAPK→ JAK→ hBD3	[59]
HGFR/c-Met	Differentiation, migration, mitogenic activity, invasive growth, wound healing	◾ Interacts with CagA	[51]
		◾ Induces cell migration and autophagy	[51,52]
		◾ Induces proliferation of primary cells	[53,54]
		◾ Promotes invasive growth via MMP activation	[55]
		◾ Leads to c-Met ectodomain shedding by *Hp*-induced ADAM10	[57]
FGFR	Angiogenesis, chemotaxis, differentiation, migration, embryonic development	◾ n.d.	
PDGFR	Angiogenesis, chemotaxis, differentiation, migration, survival, wound healing	◾ n.d.	
VEGFR	Angiogenesis, chemotaxis, differentiation, migration, survival, wound healing	◾ n.d.	
c-Kit	Hematopoiesis, survival, proliferation	◾ n.d.	
**nRTKs**	**Function**	**Role in *Hp* pathogenesis**	**References**
Abl family	Apoptosis, differentiation, migration, proliferation	◾ CagA kinase	[60,61,62]
		◾ Implicated in cell elongation and migration	[60,61,62]
		◾ Promotes cell death in B cells	[63]
		◾ Acts as switch between apoptosis and cell survival in epithelial cells	[64]
		◾ Inhibits EGFR endocytosis and limits EGFR turn-over	[65,66]
JAK	Immunity, cell division, cell death, tumor formation	◾ Leads to *c-myc* transactivation and enhanced cell migration	[67]
		◾ Involved in the synthesis of hBD3	[59,68]
		◾ Involved in NF-κB-mediated IL-8 expression	[69]
FAK	Adhesion, cytoskeleton rearrangements, migration	◾ Activated by *Hp*/integrin-β1 interaction	[28]
		◾ Promotes cytoskeleton rearrangement, cell motility, scattering	[28,41,42]
Src family	Adhesion, cytoskeleton rearrangements, growth, RTK signaling	◾ Activated by *Hp*/integrin-β1 interaction	[28]
		◾ CagA kinase	[60,61,62]
		◾ Implicated in cell elongation and migration	[60,61,62]
		◾ Promotes cell death in B cells	[63]

^1^ Abbr.: *Hp*, *H. pylori*; n.d., not determined.
